# The Use of RNAi Technology to Interfere with *Zfx* Gene Increases the Male Rates of Red Deer (*Cervus elaphus*) Offspring

**DOI:** 10.1155/2020/9549765

**Published:** 2020-05-18

**Authors:** Limin Wei, Jifeng Xi, Yongsheng Zhang, Bo Zeng, Feng Li, Kaisheng Wang, Siyuan Zhang, Xitang Zhao, Ying Li, Hong Shen, Song Jiang, Bin Jia

**Affiliations:** ^1^College of Animal Science and Technology, Shihezi University, Shihezi 832003, China; ^2^Farm Animal Genetic Resources Exploration and Innovation Key Laboratory of Sichuan Province, Sichuan Agricultural University, Chengdu, Sichuan 611130, China; ^3^Key Laboratory of Southwest China Wildlife Resources Conservation (Ministry of Education), China West Normal University, Nanchong, Sichuan 637002, China; ^4^College of Biology and Pharmacy, Yulin Normal University, Yulin, Guangxi 537000, China; ^5^Xinjiang Production and Construction Corps Second Division 34, Kuerle, Xinjiang 841000, China

## Abstract

Zinc finger protein X-linked (*Zfx*) was regarded to be a sex determination factor and plays a critical role in spermatogenesis. RNAi is an effective method of silencing *Zfx* mRNA expression. However, there has been little research on the use of RNAi technology to control the sex of the offspring of red deer (*Cervus elaphus*). The objective of this study was first to explore an efficient method to alter the red deer offspring sex-ratio by silencing the gene *Zfx* during spermatogenesis. Three recombinant expression vectors pLL3.7/A, pLL3.7/B, and pLL3.7/C were constructed to interrupt the *Zfx* gene. The results showed that the expression of *Zfx* mRNA was significantly silenced by pLL3.7/A (*P* < 0.01), compared with the control group. The group injected with pLL3.7/A produced 94 red deer, including 68 males and 26 females. The male rates (72.34%) were significantly higher than the control groups (*P* < 0.01). Our result suggests that *Zfx* siRNA is a useful approach to control offspring sex in red deer. This study further confirms that the *Zfx* gene plays a significant role in the process of X spermatogenesis.

## 1. Background

The red deer (*Cervus elaphus*) is the second-class protected animal in China and an important economic animal. It has a high output of antlers and great economic value. Antler is a kind of precious traditional Chinese medicine and a valuable nourishing health product [1, 2]. The velvet antler is considered to have various pharmacological activities, such as immunomodulatory, wound-healing effects, anti-inflammatory, and enhancement of sexual function [2–4]. The main purpose of raising red deer is to obtain antler, and only the male produces antler, so the producers have a great interest in obtaining more male fawns for the economic benefits of red deer breeding. Therefore, a technology is urgently needed in the future to control the sex of the red deer and improve the male ratio, which is an important means to increase the production of antlers and increase economic benefits.

The sex of the offspring of diploid vertebrates depends on the sex chromosome of the male parent [5]. The struggle between X and Y haploid sperm exists not only in the ovary but also in the testis [6]. Most of the current efforts in gender determination are focused on segregating X and Y spermatozoa according to physical parameters, such as density, swimming velocity, surface charge, and sex-specific antigens. Up to now, flow cytometry (FCM) analysis has been the most accurate method for sperm separation, in which accuracy can be reached up to 90% [7]. While success using sorted sperm can be achieved, it is quite costly and beyond the expertise of most production facilities. We hypothesized that controlling the crucial specific mRNA of X and Y sperms may be an effective method to determine the sex of animals at a low cost.

*Zfx* and *Zfy* are thought to play a major role in sex determination during spermatogenesis [8–11]. The binding of zinc finger protein motifs to nucleic acid binding proteins plays an important role in the sex determination of early embryos [12, 13]. Comparing the amplification patterns of *Zfx*/*Zfy* genes is a simple and accurate method for animal sex identification, which has been currently widely used [14–16]. The full-length *Zfx* protein is encoded on the X chromosome and contains a nuclear localization sequence (NLS), an acidic transcriptional activation domain (AD), and a DNA-binding domain (DBD) consisting of 13 C2H2-type zinc fingers [17]. *Zfx* is concerned with X spermatogenesis, which can serve as a strong transcription activation factor, and guides the target genes positioning in X sperm nuclei through the nuclear membrane [18]. Zhang and colleagues [19] found that *Zfx* played an important role in the process of X sperm development. Silencing the expression of *Zfx* can interfere with the normal function of these sex-specific genes after meiosis I [19].

RNAi is an effective method for gene silencing, which could effectively make target genes lose functions [20–22]. Using RNAi to silence the expression of *Zfx* mRNA to restrict or deteriorate the development of haploid X sperm is an effective way to skew the sex ratio of offspring towards males [19]. However, there has been little research on the use of RNAi technology to control the sex of the offspring of red deer. Therefore, in this study, specific RNA interference fragments were first designed to interfere with the development of X sperm, in order to change the male proportion of red deer offspring and improve the market economic benefits of the red deer industry in the world.

## 2. Materials and Methods

### 2.1. Experimental Animals

Healthy red deer were selected from the Xinjiang Production and Construction Corps Second Division 34, including16 stags and 216 does. Among them, we randomly selected 8 stags and 108 does as an experimental group and the rest as a control group. All experimental animals were fed under the same conditions. All procedures used in this experiment were reviewed and approved by the Animal Care and Use Committee of Shihezi University. All red deer care and use were conducted in strict accordance with the Animal Research Committee guidelines of Shihezi University. All orchiectomy surgery was performed under anesthesia with 4 mL Xylazine Hydrochloride Injection (100 mg/mL) (Qingdao Hanhe Animal and Plant Medicine Co., Ltd., product number 171225) per deer and resuscitation with 8 mL Nikethamide Injection (250 mg/mL) (Qingdao Hanhe Animal and Plant Medicine Co., Ltd., product number 171106) per deer according to the manufacturer's instructions, and all efforts were made to minimize suffering. This study of injection plasmid did not cause any health effects to the animals. After injection, animals were returned to their standard housing.

### 2.2. Construction of PLL3.7-shZfx Recombination Vectors

*Zfx* siRNA sequences were evaluated and selected by red deer *Zfx* sequence (KP257294.1) and siRNA design principles [23, 24]. According to the design principle on siRNA, preliminary screening of the designed siRNA sequences was processed, and then the siRNA sequences were selected and compared the homology with red deer *Zfy* sequence (MN560153) through BLAST. Nonspecific fragments were eliminated, and 3 specific siRNA sequences were finally selected (Table 1). The restriction endonucleases (*HpaI* and *XhoI*) were added to the 5′ and 3′ ends of the siRNA sequence. The sense and antisense strands of each oligonucleotide sequence (Table 2) were annealed. The annealed product was connected to pentilox 3.7 (pLL3.7) cut by *HpaI* and *XhoI* (Takara, Biotechnology Co., Dalian, China). The lentiviral transfer vector pLentiLox3.7 (pLL3.7) contains a mouse U6 RNA pol III promoter [1]. In addition, *HpaI* and *XhoI* restriction enzyme, bacterial resistance ampicillin, and selectable markers GFP were found on the vector, which was convenient for the resistance screening of transformed or transfected positive cells. The recombinant siRNA expression vectors were named as pLL3.7/A, pLL3.7/B, and pLL3.7/C and then transformed into E. coli dh5*α* active cells (Tiangen Biotech Co., Beijing, China). The plasmids were extracted from the bacterial solution using TIANprep Mini Plasmid Kit (Tiangen Biotech Co., Beijing, China) and stored at -20°C.

## 3. *Zfx* Gene RNAi Experiment In Vitro

### 3.1. Isolation and Culture of Germ Cells

The improved two-step enzymatic digestion method was used to isolate germ cells of the red deer testis [25]. The spermatogenic cells were cocultured with Sertoli cells in vitro in a CO_2_ incubator [26, 27]. The cells were cultured in 6-well cell culture plates, and each well contained 3 × 10^5^-6 × 10^5^ germ cells in an incubator at 37°C temperature, 5% CO_2_, and 95% relative humidity.

### 3.2. Transfection of *Zfx* RNAi Vector and qRT-PCR Analysis

After culturing 24 h, reconstructed vectors were transfected into cultured cells using Lipofectamine 3000 (Invitrogen, USA). The nutrient solution was changed, and the cells were observed under Lipofectamine 3000 every 24 h during culturing. In this experiment, the expression of the fluorescent protein was observed by a fluorescence inverted microscope after transfection for 24 h, 48 h, 58 h, and 72 h, respectively. The highest transfection efficiency of cells RNA was extracted by Tiangen total RNA extraction reagent and then reverse-transcribed into cDNA by RT reagent kit (Takara).

### 3.3. The RNAi Efficiency of Each Vector In Vitro

The mRNA level of *Zfx* and *Gapdh* (Table 3) was determined by RT-qPCR (LightCycler2.0, Roche, Basel, Switzerland). The RT-qPCR reaction system contained 10 *μ*L SYBR@ Premix Ex TAqTM (2×), 0.4 *μ*L oligonucleotide primers, 2 *μ*L template, and sterile distilled water up to 20 *μ*L. And the program was as follows: initial denaturation step (30 s at 95°C), 40 cycles of 95°C for 10 s, 60°C for 30 s, and 72°C for 30 s. Each sample was replicated three times to increase the degree of accuracy.

## 4. *Zfx* Gene RNAi Experiment In Vivo

### 4.1. The Injection of Plasmid in the Testis of Red Deer

Testicular injection technology is an important technical means of animal genetic engineering and embryo engineering. The testicular injection includes three methods: rete testicular injection, seminiferous tubule injection, and testicular interstitial injection, in which rete testicular injection can efficiently obtain positive offspring carrying the target gene [28]. Our laboratory successfully used testicular injection combined with RNAi interference technology to significantly change the sex of offspring [19, 29–31]. In this study, red deer were injected into rete testicular through the vertical axis by an injector after general anesthesia with Xylazine Hydrochloride. In the experimental group, the recombinant plasmid expression vector was diluted with PBS containing Penicillin-Streptomycin, then injected into 8 stags' testis at 3 mg per stag (1.5 mg in each testis), for 4 times in 10 days. Three days after the fourth injection, the 8 stags were randomly put into 8 female breeding enclosures (9-15 does in each breeding enclosures) for natural mating. The control group was normal stag without the injection of RNAi vectors. During the whole experiment, all the conditions were the same.

### 4.2. Statistical Analysis

Data were analyzed by SPSS Statistics 20.0 (IBM Corp.). The quantitation results were analyzed by using *Gapdh* expression as an internal standard and the double standard curve. The difference in expression of mRNAs was calculated using equation 2ΔΔCt, where ΔCt = Ct (*Zfx*) − Ct (*Gapdh*) and ΔΔCt = ΔCt (*Zfx*)ΔCt (control). The significant difference of differences in mRNA levels was quantified by one-way ANOVA (LSD). The significance of differences between the male and female proportion of offspring was assessed using the *χ*^2^ test. Figures were constructed by GraphPad Prism 8.0.1.

## 5. Results and Discussion

### 5.1. Results

#### 5.1.1. Germ Cell Separation and Transfection

**Figure 1:**
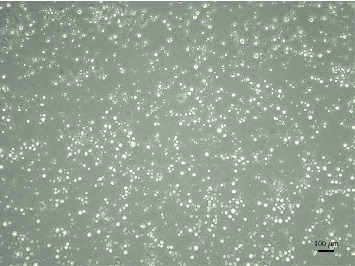
Separation of red deer germ cells. Germ cells were round and full in shape with a clear outline and uniform size, and the isolated Sertoli cells were irregular; most of them were polygonal and triangular. The cells were observed under the inversion fluorescence microscope (LeicaDMi8, Leica Microsystems, Wetzlar, Germany). Scale bars represent 100 *μ*m.

**Figure 2:**
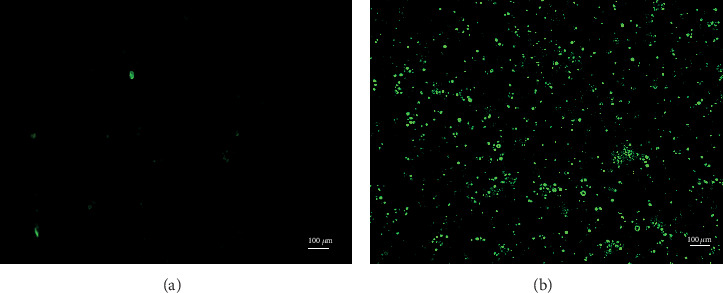
Transfection interference vector 48 h and 58 h of germ cells. *Zfx* gene RNAi vector was transferred into red deer germ cells, and the fluorescent labels were transfected germ cells. (a) There was some fluorescence expression at 48 h. (b) Fluorescence expression was the highest at 58 h. The cells were observed under the inversion fluorescence microscope (LeicaDMi8, Leica Microsystems, Wetzlar, Germany). Scale bars represent 100 *μ*m.

Collagenase IV, hyaluronidase, and trypsin were used to digest the testicular tissue of red deer into germ cells and Sertoli cells; the segregated germ cells were round and full in shape with a clear outline and uniform size. However, the isolated Sertoli cells were irregular; most of them were polygonal and triangular (Figure 1). After culturing for 24 h, reconstructed vectors were transfected into cultured cells. There was no fluorescence expression at 24 h after transfection; however, there was some fluorescence expression at 48 h, and the highest fluorescence expression was at 58 h; moreover, the transfection efficiency could reach about 90%. However, at 72 h, the viability of the cells decreased, and some cells showed apoptosis. So after transfection for 58 h, the cell mRNA was extracted and then reverse-transcribed into cDNA. The results of transfection at 58 h showed that the *Zfx* gene RNAi vector was successfully transferred into germ cells of the red deer (Figure 2).

## 6. The Expression of *Zfx* mRNA in Injection Groups and Control Groups by qRT-PCR

**Figure 3:**
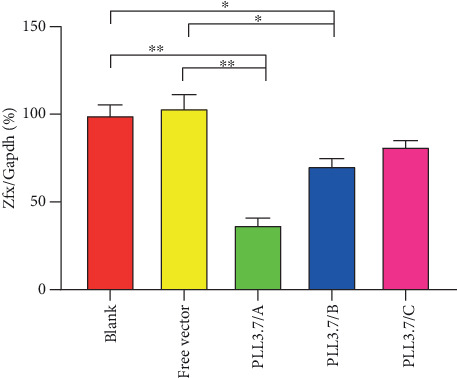
mRNA expression level of *Zfx*. *Zfx* mRNA expression was normalized against that of *Gapdh*, with expression in the control group. Data are the mean ± sd. ^∗^*P* < 0.05 and ^∗∗^*P* < 0.01 compared with the control.

The *Zfx* mRNA level results showed that all recombination vectors were lower than the control groups which were the blank and vector-free groups (Figure 3). Specifically, the *Zfx* mRNA levels of test groups injected with pLL3.7/A (*P* < 0.01), were significantly lower than control groups and transfected pLL3.7/B were lesser than the control group (*P* < 0.05). Therefore, the pLL3.7/A vector was selected for use in the in vivo experiment because it had the least expression of *Zfx* mRNA (*P* < 0.01).

## 7. Statistical Analysis Sex Proportion of the Offspring

The gestation period of red deer is 246 ± 7 days, with 1 litter per fetus. The noninjection group, as the control group, produced 98 red deer, including 50 males and 48 females, while the group injected with pLL3.7/A produced 94 red deer, including 68 males and 26 females. The male rates (72.34%) were significantly higher than the control groups (*P* < 0.01) (Table 4).

This result suggests that the *Zfx* gene plays an important role in the process of X sperm formation. *Zfx* siRNA is an advanced method to control red deer X spermatogenesis and sex ratio of offspring.

Conception rate means the no. of pregnancies/no. of all females; reproductive rate means litter per pregnancy; stillbirth rate means the no. of stillborn fetus/no. of pregnancies. ∗∗ means the extremely significant difference between the noninjection group and treatment groups (*P* < 0.01).

## 8. Discussion

Although the reproduction of the sex determination mechanism requires multiple gene interactions, the sex ratio in nature is stable at 1 : 1 [32]. The formation of X or Y sperm during spermatogenesis plays an important role in the sex determination of offspring [33]. Previous studies in our laboratory found that *Zfx* and *Zfy* siRNA can change the sex ratio of animal offspring, such as cows, mouse, and sheep [19, 30, 31]. Red deer is a special economic animal, and its antler has great market economic value. However, there has been little research on the use of RNAi technology to control the sex of the offspring of red deer. In our study, the red deer *Zfx* siRNA was first designed to change the sex ratio of the offspring, once again demonstrating the important role of *Zfx* in gender. More importantly, it is of great significance to increase the male ratio of red deer offspring to increase antler yield and economic benefits.

At present, there is only a little research on *Zfx* gene silencing to make the sex shift of offspring, so the mechanism of action of *Zfx* in sperm is not clear. Some studies have shown that reducing the level of *Zfx*/*Zfy* mRNA expression will make the physiological function of X/Y sperm worse, which can influence the offspring gender [19, 29–31, 34]. *Zfy* controls sperm head and tail formation and neck development [35], which is highly expressed between meiosis I and meiosis II. It can regulate sperm morphology and ROSI efficiency and promotes second meiosis [36, 37]. Similarly, *Zfx* gene is female-specific after meiosis I and is always expressed throughout spermatogenesis [18]. The high expression of *Zfx* in round sperm cells of adult mouse testis controls the self-renewal of spermatocytes. Generally speaking, *Zfy* gene is to ensure the occurrence of the second meiosis, and the X-related gene *Zfx* on the chromosome has a promoting effect [38]. Reducing the expression level of *Zfx* mRNA will affect the development of haploid X sperm, which will lead to a decrease of female offspring [19].

However, there was no health abnormality and decreased reproductive performance in the experimental animal group, compared with the control animal group. Then, male red deer were placed in female breeding enclosures for natural mating within 10-40 days after the fourth injection of the vector. This is in line with the sheep's spermatogenic cycle and spermatogenic wave [39] in which *Zfx* homology is closest to red deer. According to our laboratory experience, four injections of interference fragments will generally produce the effect of 45 days, and there is no effect beyond 45 days, 10-30 days after injection is the best matching time. In addition, the fertility rate, dystocia rate, abortion rate, reproduction rate, empty embryo rate, offspring survival rate, and offspring deformity rate of the female deer experimental group were not abnormal compared with the control group. In conclusion, under the same feeding conditions, compared with the normal control group, the sex ratio of offspring in the experimental group shifted significantly, and the male ratio reached 72.34%. It indicated that the injection of interfering fragments changed the sex ratio of offspring without effecting the physiological function and reproductive performance of animals. However, with the increase of male deer, it seems that this may also cause some production problems for a long time; for example, the less female deer will also lead to fewer offspring. But the number of female deer in our farm is large enough, so it will not affect the offspring because of injection. Additionally, in general, the deer farms screen and retain sufficient female deer with strong reproductive capacity every year, so it will not reduce the number of offspring because of sex control. Therefore, the silencing of the *Zfx* is safe and effective for the future application of sex control.

Spermatogenesis is a highly regulated and orderly process. It is regulated by many genes, but its mechanism is still unclear [40, 41] and further research is needed. However, this study, the *Zfx* gene silencing technology was successfully used to significantly increase the number of male red deer in the offspring, which not only increased the economic benefits in production but also provided the theoretical reference for the subsequent research on gender control mechanism in scientific research.

## 9. Conclusion

The results of this study indicate that the use of RNAi to interrupt *Zfx* is a useful approach to control red deer sex and further confirms that the *Zfx* gene plays a significant role in the process of X spermatogenesis.

## Figures and Tables

**Table 1:** *Zfx* cDNA templates for siRNA in vitro transcription.

Zfx (KP257294.1)	
A	GTGAGGACTACCTTATGAT
B	GACGTCTTAGCTTCTGATA
C	GAAGCAGATGTATCTGAAA

**Table 2:** *Zfx* RNAi oligonucleotide sequence with *HpaI* and *XhoI* enzyme sites.

Vector shRNA	Oligonucleotide sequence
pLL3.7 A	F: 5′-TGTGAGGACTACCTTATGATTTCAAGAGAATCATAAGGTAGTCCTCACTTTTTTC-3′
	R: 5′-TCGAGAAAAAAGTGAGGACTACCTTATGATTCTCTTGAAATCATAAGGTAGTCCTCACA-3′
pLL3.7 B	F: 5′-TGACGTCTTAGCTTCTGATATTCAAGAGATATCAGAAGCTAAGACGTCTTTTTTC-3′
	R: 5′-TCGAGAAAAAAGACGTCTTAGCTTCTGATATCTCTTGAATATCAGAAGCTAAGACGTCA-3′
pLL3.7 C	F: 5′-TGAAGCAGATGTATCTGAAATTCAAGAGATTTCAGATACATCTGCTTCTTTTTTC-3′
	R: 5′-TCGAGAAAAAAGAAGCAGATGTATCTGAAATCTCTTGAATTTCAGATACATCTGCTTCA-3′

Notes: using pLL3.7 as the vector construction of shRNA interference fragment.

**Table 3:** Conditions of PCR and oligonucleotide primer pairs.

Target *gene*	Gene bank	Sequence of primer (5′→3′)	Annealing temperature	Product (bp)
*Zfx*	KP257294.1	F: TATGGATTCACTCGTCAA	60°C	120
		R: CTCAGATGTAACAGAAGAAG		
*GAPDH*	AY650282.1	F: AAGGCCATCACCATCTTCCA	60°C	80
		R: CCAGCATCACCCCACTTGA		

**Table 4:** The number of female and male offspring in each group of red deer.

Subgroup	Conception rate	Reproductive rate	Stillbirth rate	Offspring birth weight	Female	Male	Male rate
Noninjection	100/108	1	2/100	10.65 kg	48	50	51.02%
Injection	97/108	1	3/97	10.73 kg	26	68	72.34%^∗∗^

## Data Availability

Red deer *Zfx* sequence and red deer *Zfy* sequence are, respectively, archived at NCBI GenBank under accession numbers KP257294.1 and MN560153.
